# Do Termitaria Indicate the Presence of Groundwater? A Case Study of Hydrogeophysical Investigation on a Land Parcel with Termite Activity

**DOI:** 10.3390/insects11110728

**Published:** 2020-10-24

**Authors:** Jamilu Bala Ahmed, Abdullahi Salisu, Biswajeet Pradhan, Abdullah M. Alamri

**Affiliations:** 1 Department of Geology, Faculty of Science, Federal University Lokoja, Lokoja 1154, Nigeria; senjamil32@gmail.com; 2 Department of Biological and Agricultural Engineering, Faculty of Engineering, Universiti Putra Malaysia, Serdang 43400, Malaysia; abdullahiskiru@gmail.com; 3 Centre for Advanced Modelling and Geospatial Information Systems (CAMGIS), School of Information, Systems & Modelling, Faculty of Engineering & IT, University of Technology Sydney, Sydney, NSW 2007, Australia; 4 Department of Energy and Mineral Resources Engineering, Seiong University, Choongmu-gwan, 209 Neungdong-ro, Gwangjin-gu, Seoul 05006, Korea; 5 Earth Observation Center, Institute of Climate Change, Universiti Kebangsaan Malaysia, Bangi 43600 UKM, Selangor, Malaysia; 6 Department of Geology and Geophysics, College of Science, King Saud University, P.O. Box 2455, Riyadh 11451, Saudi Arabia; amsamri@ksu.edu.sa

**Keywords:** termitaria, termites, groundwater, VES, GIS

## Abstract

**Simple Summary:**

Termitaria are nests of termites built from clayey soils. These nests can protrude to several meters high above the ground-surface. They serve not only as habitats for termites but also as foraging hotspots for grazing and browsing animals due to an increase in nutrient recycling that results in the growth of nutrient-rich vegetation around termitaria. There have been suggestions that a relationship exists between termitaria and groundwater due to the high demand of water by termite colonies for their nest building, to maintain high humidity and for their metabolism. However, few studies are available to directly or indirectly indicate this relationship. In this study, effort was made to investigate the direct relationship between termitaria and groundwater and to further answer the question on whether termitaria can indicate the presence of groundwater. A small parcel of land with a termitarium was selected for this study where nine resistivity soundings were made with one at the foot of the termitarium. The result obtained indicated a suitable geoelectrical signature around the termitarium and the integration of six geoelectrical parameters revealed the termitarium as falling in the middle of the most suitable zone of groundwater and hence can be used as a biomarker.

**Abstract:**

Termite nests have long been suggested to be good indicators of groundwater but only a few studies are available to demonstrate the relationship between the two. This study therefore aims at investigating the most favourable spots for locating groundwater structures on a small parcel of land with conspicuous termite activity. To achieve this, geophysical soundings using the renowned vertical electrical sounding (VES) technique was carried out on the gridded study area. A total of nine VESs with one at the foot of a termitarium were conducted. The VES results were interpreted and assessed via two different techniques: (1) physical evaluation as performed by drillers in the field and (2) integration of primary and secondary geoelectrical parameters in a geographic information system (GIS). The result of the physical evaluation indicated a clear case of subjectivity in the interpretation but was consistent with the choice of VES points 1 and 6 (termitarium location) as being the most prospective points to be considered for drilling. Similarly, the integration of the geoelectrical parameters led to the mapping of the most prospective groundwater portion of the study area with the termitarium chiefly in the center of the most suitable region. This shows that termitaria are valuable landscape features that can be employed as biomarkers in the search of groundwater.

## 1. Introduction

Termites are insects of enormous ecological, economic and medicinal importance [1,2,3]. Their habitats although highly varied, range from being subterranean, epigeal to arboreal [4]. The epigeal nests which protrude from the ground to rise several meters high are without a doubt the most common form of termite nests generally referred to as termitarium or termite mounds. Termitaria are long-lasting structures that are built from clays and can stand for several centuries [5]. Built in a wide variety of shapes and sizes, a single termitarium can house several thousands to millions of termite colony members [6], where such locations become activity hotspots for many foraging, browsing and even grazing animals as a result of termites’ reworking of the surrounding soil to modify the physical, chemical and biological properties such as soil porosity, soil pH, soil organic matter, water infiltration capacity, etc. [7,8].

The first impression that comes to our mind about termites is the considerable damage they cause to man-made wooden structures and agronomic and forest resources [9], which is globally estimated to cost around USD 40 billion [10]. Few however are informed about the many benefits derivable from termites and their termitaria which can be sustainably managed to human advantage. Pharmaceutically, termitaria soils are consumed for regulating stomach pH while the termites themselves are used as alternative treatments for asthma, hoarseness and pregnancy complications [3]. Termitaria soils facilitate the growth of woody vegetation and contribute to the maintenance of savannah biodiversity just as they are appropriate sampling media for the exploration of concealed metallic mineralization such as gold, silver, copper, zinc, uranium, etc. [11].

The relationship between termites or termiteria (conspicuously above-ground) and groundwater (several depths below-ground) have been sparsely investigated. Few studies have revealed how termites depend on groundwater supplies for metabolism [12,13,14,15] since proximity to surface water (streams and rivers) can be threatening to the termite colony when floods occur [8,16,17]. Using a combination of field survey and geographic information system (GIS) analyses, Ahmed II et al. [18] revealed how termitaria density, height and, to some extent, basal diameter are controlled by piezometric surfaces. In a related work, groundwater control factors as identified from field studies and remotely sensed data such as static water level, groundwater fluctuation, lineament density and intersection as well as slope were found to have strong to moderate relationships with certain categories of termiteria [19]. Furthermore, the use of geophysical exploration techniques in probing the subsurface for an examination of groundwater conditions on and off termitaria locations was employed by [20]. The result showed that most aquifers surrounding termitaria had greater groundwater productivity potential compared to those areas away from termitaria and hence termitaria locations could be more favourable spots for sinking groundwater abstraction structures.

This paper is therefore, a follow up on a previous study [20] with the aim of investigating the most favourable spots for locating groundwater structures (boreholes) on a small parcel of land with conspicuous termite activity earmarked for development. There is general scarcity of reference material on this subject matter, so therefore there is a need to synthesize the little existing knowledge to provide new insights into the termite–groundwater relationship. The usual practice in this part of the world is for few geophysical soundings (2–3 points) to be made where the best is selected for drilling. In the case of this study, several soundings were made with one of the sounding points located at the foot of the termitarium to aid comparison and the selection of best points.

## 2. Materials and Methods

### 2.1. Study Area

**Figure 1:**
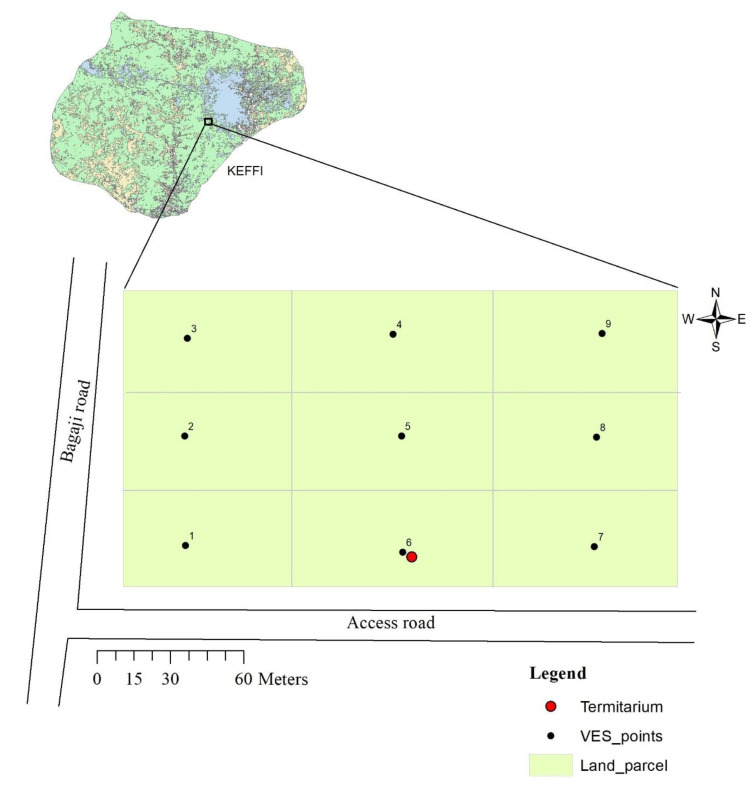
Map of the study area showing the location of termitarium and vertical electrical sounding (VES) points.

**Figure 2:**
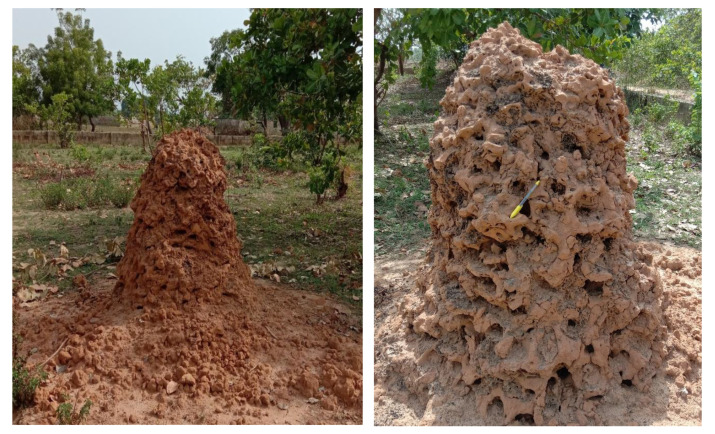
Termitarium in the study area partly destroyed by human activity.

The study was conducted on a 200 ft × 100 ft parcel of land located in Keffi District of Nasarawa State, Nigeria (Figure 1). The land has been left undeveloped and uncultivated for a long period and consequently has been covered by shrubs and few trees. There are in all 6 cashew trees (*Anacardium occidentale*), 3 neem trees (*Azadirachta indica*) and 2 locust beans trees (*Parkia biglobosa*). Whether these trees were intentionally planted remains unresolved. The study area is covered by reddish lateritic soil and underlain by rocks of schistose lithology. Evidence of termite activity is visible with the presence of a high-rising termiteria originally measuring up to 2.5 m in height (as was first measured in 2017) with a basal diameter of about 1.45 m. The shape of the termiteria is conical and was built by the termite genus *Macrotermes* (Figure 2). Tropical climatic condition characterize the study area with distinct rainy and dry seasons. Groundwater levels vary in response to topography, being shallow around valleys and depressions and deep around high grounds. Further, description of the climatic, topographical as well as the human activities in the study area (Keffi) can be found in [21].

### 2.2. Geoelectric Data Acquisition

The ABEM SAS 1000 resistivity meter was deployed to the site for resistivity sounding using the renowned 1D Vertical Electrical Sounding (VES) method and employing the conventional Schlumberger electrode array method. The study site was gridded into 9 blocks from where sounding was conducted on each block making a total of 9 VESs. The current electrode separation (AB) range was from 1 to a maximum of 300 m while the potential electrode separation (MN) varied intermittently between 0.5 and 10 m to enable a suitable depth penetration.

The VES field data acquired were interpreted using a 1D inversion iteration program, WinRESIST 1.0, through the following steps:

1. Field data smoothing, removing electrical noises and matching the generated smoothed field data curves with standard auxiliary curves (e.g., [22]).
2. Initial geoelectrical model preparation specifying number of layers, their thicknesses and corresponding resistivity and incorporating the geological and well information of the study area (e.g., [23]).
3. Computer iteration to reach the best fit between the smoothed field curve and the calculated one. Thirty (30) iterations were performed on each dataset to produce a root mean square (RMS) error of less than 10% (e.g., [24]).

### 2.3. Geoelectric Parameters

The acquired VES field data were subjected to processing to aid the interpretation. The final VES interpretation results produced primary geoelectric parameters—namely, layer thickness (*h*_i_) and layer resistivity (*ρ*_i_)—from where other important parameters were derived which include: weathered basement (aquifer layer) thickness and resistivity and overburden thickness. Furthermore, the primary geoelectric parameters were used to generate secondary geoelectric parameters (Dar-Zarrouk)—namely, total longitudinal conductance (*S*), total transverse resistance (*T*) and coefficient of anisotropy (λ)—which according to [25,26] are important parameters in evaluating the properties of an aquifer. Calculating these parameters involves the use of relevant equations derived by [27].

Total longitudinal conductance (*S*) is given as:

*S* = *h*_1_/*ρ*_1_ + *h*_2_/*ρ*_2_ + …+ *h*_n_/*ρ_n_* (1)

Total transverse resistance (*T*) is given as:

*T* = *h*_1_*ρ*_1_ + *h*_2_*ρ*_2_ + … + *h*_n_*ρ*_n_ (2)

Coefficient of anisotropy (λ) is given as:

λ = (ρ_t/_ρ_L_)^1/2^ = (*TS*/H^2^)^1/2^

where ρ_t_ is average transverse resistivity and is given by Equation (3):

ρ_t_ = T/H = (Σh_i_ ρ_i_)/Σh_i_ (3)

ρ_L_ = average longitudinal resistivity and is given by Equation (4):

ρ_L_ = H/S = Σ h_i_/(Σh_i_/ρ_i_) (4)

where *ρ* is resistivity of a layer and *h* is the thickness of the layer.

### 2.4. Groundwater Potential Evaluation

**Figure 3:**
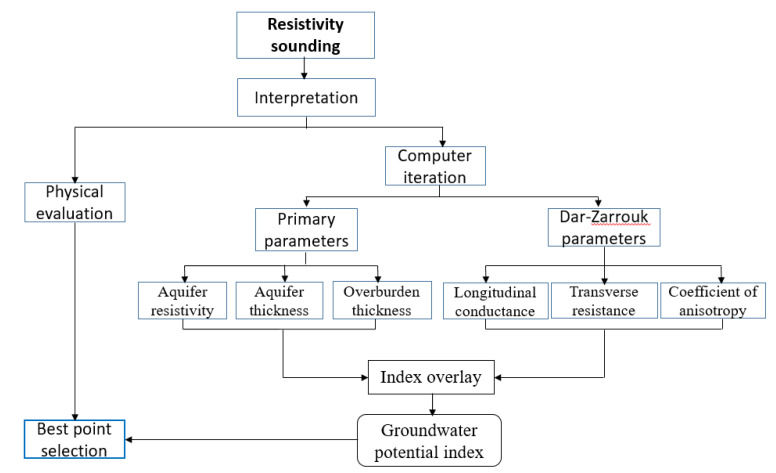
Methodology flowchart.

To evaluate the potential of the VES points and, consequently, the entire study area, two different approaches were used. In the first approach, three geophysicists were asked to physically examine the field VES curves and rank them based on their potential for groundwater. Borehole drillers in this part of the world often rely on a physical interpretation of field VES curves to select drill sites with reasonable success rates as allowing more time for proper interpretation is a burden on them especially when dealing with ignorant clients. In the second technique, thematic maps of the primary and secondary geoelectric parameters were produced in the ArcGIS 10.7 environment using the kriging interpolation technique (e.g., [28,29]). This was followed by integration of all the thematic layers for the purpose of evaluating the groundwater potential disposition of the entire study area and selecting the best points for potential drilling success as shown in the methodology flow chart (Figure 3).

## 3. Results

### 3.1. VES Curves

**Figure 4:**
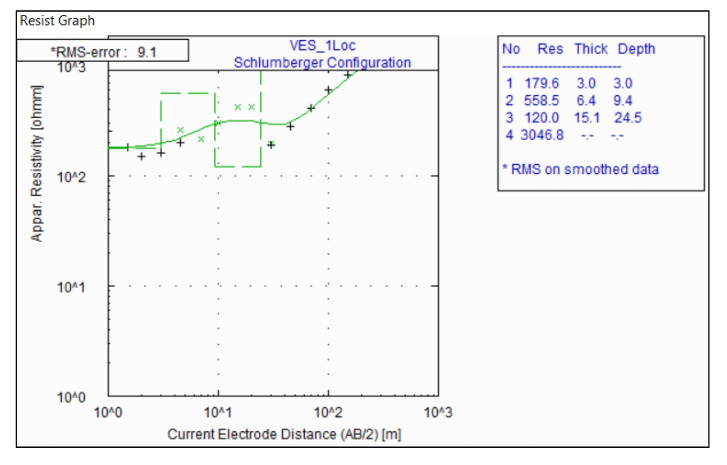

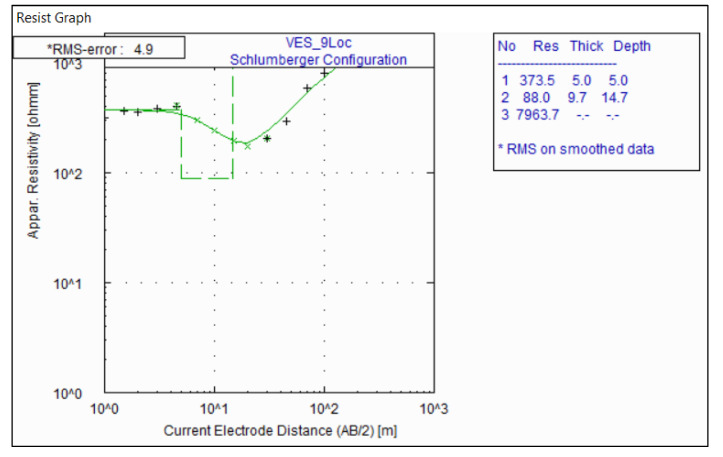
Representative VES curves in the study area.

Qualitative interpretation of the VES model curves in the study area revealed three to four geoelectrical layer conditions (Figure 4). These curve types varied from H to KH types with H being the predominant type. The characteristics of the geoelectric layers are summarized in Table 1.

### 3.2. Physical Evaluation of VES Curves

The physical evaluation of VES curves was carried out by three independent experts who are specialists in the field of geophysics. The result of this evaluation was put in a ranking order based on their potential for groundwater drilling success as shown in Table 2.

### 3.3. Primary Geoelectrical Parameters

#### 3.3.1. Aquifer Layer Resistivity

**Figure 5:**
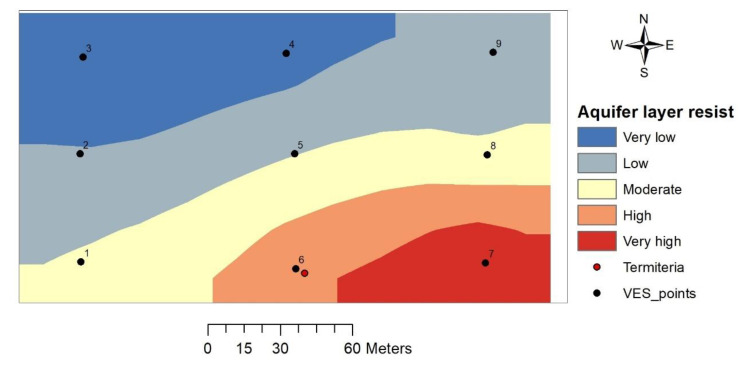
Aquifer unit layer resistivity map.

The resistivity of the aquifer unit layer (weathered basement layer) ranges between 35.4 and 243 Ωm. Thematic map of the aquifer unit resistivity showed a gradual increase in resistivity from the northwestern to southeastern part of the study area. The area surrounding the termitarium indicated resistivity values greater than 200 Ωm (Figure 5).

#### 3.3.2. Aquifer Unit Layer Thickness

**Figure 6:**
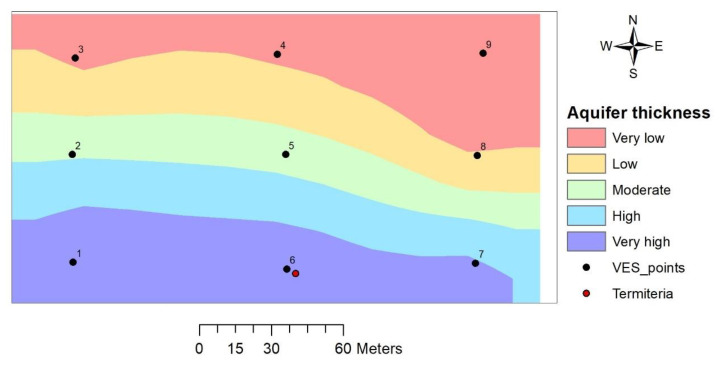
Aquifer unit layer thickness map.

The thickness of the aquifer unit (weathered layer) in the study area varies between 6.0 and 17.6 m with the highest recorded on VES 7 and the lowest on VES 8. Thematic map of the aquifer unit layer showed an increase in aquifer layer thickness from north to south (Figure 6). VES 6 where the termitarium is located falls within the region where the aquifer unit layer thickness is greatest.

#### 3.3.3. Overburden Thickness

**Figure 7:**
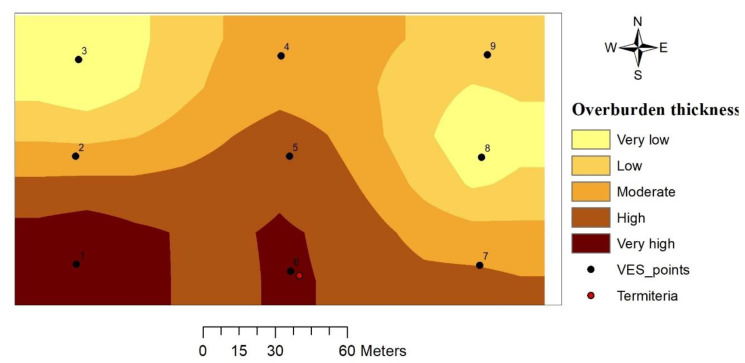
Overburden thickness map.

The sum total of subsoil and weathered rocks overlying a fresh basement rock constitutes the overburden. Thickness of overburden in the study area ranges from 8.5 to 24.5 m thick from the ground-surface. The thickness as shown from the thematic map increases in a southerly direction (Figure 7). The portions with the highest overburden thickness are represented by VES 1 and 6 (termitarium location).

### 3.4. Secondary Geoelectrical (Dar-Zarrouk) Parameters

#### 3.4.1. Total Transverse Resistance (T)

**Figure 8:**
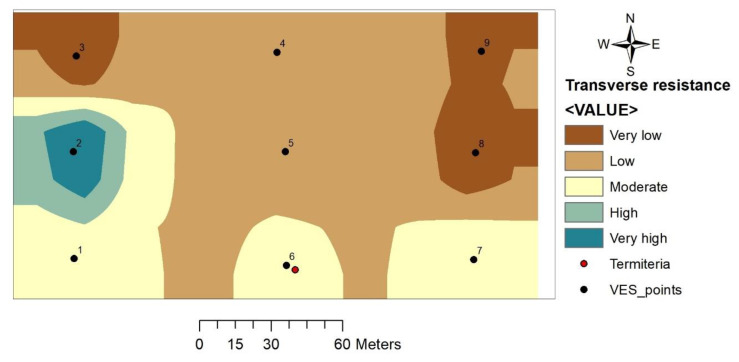
Total transverse resistance map.

Transverse resistance in the study area ranges between 1976.3 and 9308.0 Ωm^2^ with VES 2 recording the highest values while VES 3 recorded the lowest value. From the thematic map, low values of transverse resistance dominate the northern to the central part of the study area while the highest values are isolated to the southern part around VES 1, 6 and 7 (Figure 8).

#### 3.4.2. Coefficient of Anisotropy (λ)

**Figure 9:**
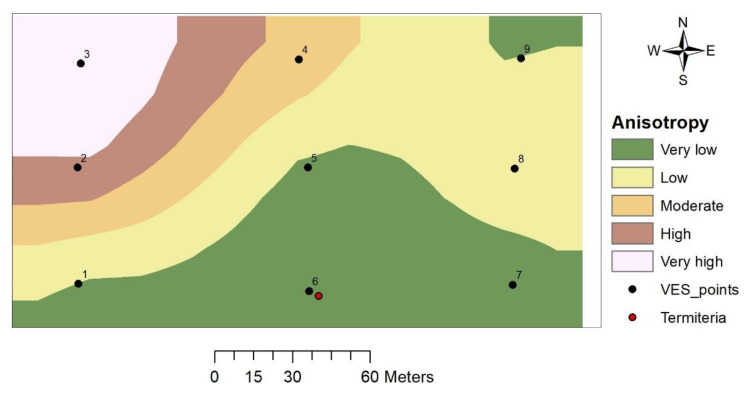
Coefficient of anisotropy map.

The coefficient of anisotropy on top of basement rock ranges between 1.08 and 2.32 with the highest value around VES 3 and the lowest around VES 6 (termitarium location). The values of the coefficient of anisotropy as depicted from the thematic map of the study area increases gradually from the northwestern to southeastern part (Figure 9), which indicates the heterogeneous and anisotropic nature of the subsurface in mainly NW–SE directions.

#### 3.4.3. Longitudinal Conductance (S)

**Figure 10:**
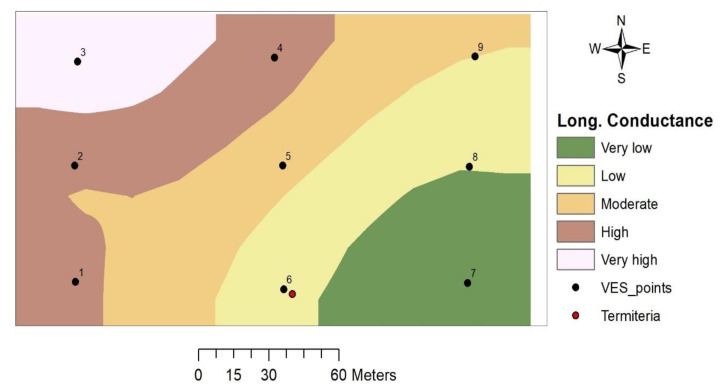
Longitudinal conductance map.

Values of longitudinal conductance in the study area range between 0.075 and 0.197 S, with the highest value recorded at VES 3 and the lowest at VES 7. The thematic map indicated a gradual increase in total longitudinal conductance values from the southeastern to northwestern portion of the study area, with the termitarium location having a low value (Figure 10).

### 3.5. Groundwater Potential Evaluation

**Figure 11:**
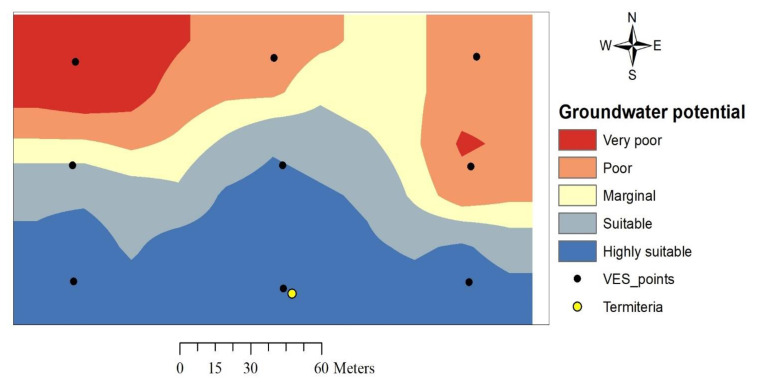
Groundwater potential map of the study area.

Evaluation of groundwater potential in this study area was achieved through the integration of all six thematic layers of aquifer unit resistivity, aquifer unit thickness, overburden thickness, total longitudinal conductance, total transverse resistance and coefficient of electrical anisotropy. The thematic layers were properly classified into five classes according to natural break classifications and assigned weightage ranging from 1 to 5 based on the suitability of a class to groundwater accumulation. The thematic layers were considered to be of equal importance as far as groundwater is concerned (no reference to suggest otherwise); hence, there was no use of weightage or hierarchy in the integration of thematic layers. The integration was carried out using the linear combination approach on a raster calculator. The final groundwater potential map was categorized into five zones using a natural break classification scheme to establish very poor, poor, marginal, suitable and highly suitable zones (Figure 11).

## 4. Discussion

### 4.1. Physical Evaluation of VES Curves

It is often the practice of drillers to utilize rapid physical assessments of VES data on the field where rigs are stationed for onward drilling. The success of this method of evaluation is dependent on the level of expertise of the geophysicists as it does not take into account many important factors such as the nature of the geology and other hydrological concerns. Another big challenge for this method is that it is highly subjective and hence largely not reproducible. Nevertheless, the result from ranking of the 9 VES points in this study, although subjective, showed a level of consistency for VES points 1 and 6 (termitarium location) to be the best and most suitable points to be considered for drilling. This means that termitaria locations would likely produce desirable electrical signatures that would be adjudged from mere physical examination as suitable for groundwater drilling purposes in the basement complex regions.

### 4.2. Primary Geoelectrical Parameters

#### 4.2.1. Aquifer Layer Resistivity

Aquifer layer resistivity is controlled by the degree of interconnected pore spaces and availability of conducting fluids [30]. Resistivity value of ≤20 Ωm for this layer is likely to be composed of highly clayey material that impedes the free flow of groundwater while values ranging between 21 and 300 Ωm indicate a variable degree of weathering—from optimum to little weathering [31,32]. However, a number of studies have shown that high groundwater yielding boreholes in the basement complex regions have aquifer layer resistivities ranging between 100 and 600 Ωm [33,34]. Based on the foregoing, the aquifer unit layer with a lower resistivity value offers better potential for groundwater and this potential is seen from the study area as decreases in a southeastern direction. This suggests that the termitarium location (VES 6) and its immediate surrounding (VES 5 and 7) are of lower groundwater potential owing to the high resistivity of the aquifer layer (107.9–243.0 Ωm).

#### 4.2.2. Aquifer Unit Layer Thickness

Aquifer layer thickness is recognized as a very important parameter for assessing groundwater potential from geophysical sounding [35]. Adeyemo et al. [30] recognized aquifer layer thickness as one of the three most important parameters after geology and aquifer layer resistivity. The thicker the aquifer layer, the larger the water column and hence the greater the potential for groundwater accumulation [22,30,36]. Aquifer layer thickness in the study area increases in a southward direction with the termitarium location and surroundings having greater thickness. From the foregoing, groundwater potential in the study area increases with increasing aquifer layer thickness which implies an increase in a southerly direction.

#### 4.2.3. Overburden Thickness

A number of studies have revealed how overburden thickness is connected with either high groundwater potential or the actual yield of boreholes (e.g., [22,36,37]). Because the overburden is no longer consolidated due to weathering and fracturing, there is an increase in pore space to accumulate groundwater and the thicker it is might also imply a thicker aquifer layer and hence larger column available for groundwater storage. From the study area, the overburden thickness is seen to increase in a southerly direction with VES 1 and 6 (termitarium location) having the highest thickness. A similar result was obtained by Ahmed II et al. [20] that led to the suggestion that termites, through bioturbation and reworking of in-situ soils are likely biological agents of weathering that add to the natural weathering process. This weathering process increases from surface to deeper levels with time, and results in the formation of thick aquifer and overburden layers.

### 4.3. Dar-Zarrouk Parameters

#### 4.3.1. Total Transverse Resistance (T)

Total transverse resistance (*T*) can serve as a measure of transmissivity of an area where high values of *T* indicate a high degree of transmissivity and consequently high groundwater potential and vice versa [38,39]. Mogaji and Lim [26] have successfully derived a relationship between transverse resistance and actual borehole transmissivity where areas devoid of any borehole or lack data on transmissivity can easily be obtained when geophysical data are available. Transverse resistance in the study area has no defined direction of increase or decrease but is dominated by low values with few isolated high- and very-low value regions. The termitarium location (VES 6) as well as VES 1 and 7 falls within the moderate value range thus indicating moderate groundwater potential.

#### 4.3.2. Coefficient of Anisotropy

Coefficient of anisotropy values in many geological terrains rarely exceed 2 [40] and are considered very useful in groundwater prospecting when the values range between 1 and 1.5 [39]. Low values of **λ** in a place corresponds to low water table fluctuations and hence have high groundwater potential [39]. The termitarium and its surroundings (VES 1, 5 and 7) falls within the very low anisotropy region with values ranging between 1.08 and 1.23. The termitarium location recorded the lowest value of **λ** and hence is expected to have the highest groundwater potential.

#### 4.3.3. Total Longitudinal Conductance

Total longitudinal conductance on the top of basement rocks is utilized in assessing the protective capacity of overburden as a natural filter for infiltrating fluids [38,41]. It is not enough to have a high potential or actual yield; the vulnerability to pollution should also be put into consideration. Longitudinal conductance has been classified as offering good protective capacity when the value is above 0.7 S, as moderate when the values range between 0.2 and 0.69 S, and as weak when the values range between 0.1 and 0.19 S [38]. The result from the study area indicated that the protective capacity of the overburden is generally weak and therefore, the groundwater is highly vulnerable to pollution with the southern part being even more at risk.

### 4.4. Groundwater Potential

The termitarium falls in the centre of the highly suitable groundwater potential region, implying that it can be utilized as a biomarker in the search for groundwater. This is because, termites require a large amount of water to make mud for termitaria building and maintenance, for their own metabolism, and to keep the nest humid and warm. This water could be easily sourced from surface locations such as streams and rivers, but termites avoid such locations for fear of inundation [20]. Therefore, locating termitaria means that half of the exploratory job has been performed at no cost [12]; however, caution must be exercised as not all termitaria (due to their diversity in sizes, regions and builder species) can be good biomarkers of groundwater [19].

## 5. Conclusions

Aquifer systems in the basement complex environment are highly complex, very localized and have a high degree of vertical and lateral inhomogeneity resulting in abstracting structures (e.g., boreholes and wells) exhibiting highly variable yields at close distances [42,43]. Most communities located within basement complex terrains are usually confronted with challenges of groundwater supply inadequacies, which can be improved by more robust investigations and the incorporation of features/structures that serve as biomarkers.

The integration of primary and secondary geoelectric parameters generated from resistivity soundings produced the groundwater potential index of the study area where the southern part is revealed to be the most suitable for siting abstracting structures. This part of the study area comprises a relatively higher aquifer layer resistivity (158.5–243.0 Ωm), higher aquifer unit layer thickness (15.1–17.6 m), deeper overburden (19.6–24.5 m thick), moderate to low transverse resistance (4549.9–5925.2 Ωm^2^) and the lowest values of coefficient of anisotropy (1.08–1.25 λ). The region also comprises low to very-low values of longitudinal conductance which means that although it has greater groundwater potential, the region is at high vulnerability to groundwater pollution.

It is interesting that the termitarium location is among the best points to be considered for drilling based on physical examination of field VES curves and also falls at the centre of the most suitable groundwater potential region. This corroborates the earlier findings of [20] in which termitaria locations were discovered to, on a regional basis, have higher aquifer layer resistivity, deeper overburden, higher infiltration rates and consequently greater groundwater potential than adjacent areas without termite activity. This further strengthens the conclusion that termites either have the ability to locate places with good groundwater prospects or are themselves agents of biological weathering, with the former being far more conceivable. Either way, this shows that termitaria are valuable landscape features that can be employed as biomarkers of groundwater by hydrogeologists in their search for groundwater.

## Figures and Tables

**Table 1:** Summary of geoelectrical layer resistivity, thicknesses, and Dar-Zarrouk parameters.

VES No.	ρ1 (Ωm)	ρ2 (Ωm)	ρ3 (Ωm)	ρ4 (Ωm)	h1 (m)	h2 (m)	h3 (m)	Σhi (m)	*S* (1/Ω)	*T* (Ωm^2^)	Λ
1.	179.6	558.5	120.0	3046.8	3.0	6.4	15.1	24.5	0.154	5925.2	1.23
2.	539.9	2905.3	104.1	5111.4	1.3	2.5	12.9	16.7	0.127	9308.0	2.06
3.	134.7	1283.2	35.4	6750.2	0.5	1.3	6.8	8.5	0.197	1976.3	2.32
4.	733.8	70.3	6724.9	-	5.2	10.5	-	15.7	0.156	4553.9	1.70
5.	413.4	107.9	5662.2	-	7.9	11.9	-	19.9	0.129	4549.9	1.22
6.	359.5	158.5	7567.8	-	7.7	13.7	-	21.3	0.107	4939.6	1.08
7.	695.7	243.0	4661.7	-	2.0	17.6	-	19.6	0.075	5668.2	1.11
8.	511.4	61.1	14,981.5	-	3.4	6.0	-	9.4	0.105	2105.4	1.58
9.	373.5	88.0	7963.7	-	5.0	9.7	-	14.7	0.124	2721.1	1.25

ρ = layer resistivity, h = layer thickness, Σhi = overburden thickness, *S* = longitudinal conductance, *T* = transverse resistance, and λ = coefficient of anisotropy.

**Table 2:** Ranking of VES based on potential drilling success.

VES Point	Curve Type	Expert 1 Rank	Expert 2 Rank	Expert 3 Rank
**1**	KH	1st	1st	2nd
**2**	KH	2nd	4th	3th
**3**	KH	4th	7th	6th
**4**	H	6th	5th	7th
**5**	H	5th	3th	5th
**6**	H	3th	2nd	1st
**7**	H	9th	6th	4th
**8**	H	7th	8th	9th
**9**	H	8th	9th	8th

Note: VES point 6 where termitaria is located emphasized with red ink.
